# CLEO closed loop framework for synthesizing medical privacy preserving tabular data

**DOI:** 10.1038/s41746-026-02999-3

**Published:** 2026-07-14

**Authors:** Siqi Wang, Jianfeng Wang, Xiaochun Cheng, Wei Zhang, Yuan Tian, Ma Ning, Fangwei Li, Yuxin Liu

**Affiliations:** 1 Taiyuan University of Technology, Taiyuan, China; 2 School of Mathematics and Computer Science, Swansea University, Swansea, UK; 3 Department of Clinical Science, Karolinska Institutet, Stockholm, Sweden; 4 Department of Medical Examination, The Central Hospital of Wuhan, Tongji Medical College, Huazhong University of Science and Technology, Wuhan, China; 5 Department of Neurosurgery, Shanxi Cardiovascular Hospital, Taiyuan, China; 6 Department of Nuclear Medicine, Hongqi Hospital Affiliated to Mudanjiang Medical University, Mudanjiang, China; 7 Department of Oncology and Hematology, Shanxi Provincial Hospital of Integrated Traditional and Western Medicine, Taiyuan, China

**Keywords:** Computational biology and bioinformatics, Engineering, Health care, Mathematics and computing

## Abstract

The sharing of patient-level structured data is strictly constrained by privacy regulations and governance, creating “data silos” that hinder multi-center research. We propose the CLEO (Clean-Learn-Evaluate-Optimize), a closed-loop framework that integrates a Gaussian Mixture Model generator with Q-learning-based optimization to formalize data synthesis as a Markov Decision Process. Experimental results on a multicenter intracranial aneurysm dataset show that CLEO achieved a combined score of 0.9232 ± 0.0124, outperforming the evaluated representative comparison methods, including TVAE, Gaussian Copula, and CTGAN. In downstream TSTR evaluation, models trained on CLEO-generated data achieved an average AUC of 0.7376, indicating that the synthetic data retained useful clinical decision signals. However, Macro-F1 results also suggest that minority-class prediction remains affected by the imbalanced class distribution. Empirical privacy auditing showed a Nearest Neighbor Adversarial Accuracy score of 0.4940, suggesting low observed re-identification risk under the adopted nearest-neighbor audit setting. These findings indicate that CLEO provides a controllable and empirically auditable framework for supporting cross-institutional research when real-world medical data cannot be directly aggregated.

## Introduction

High-quality clinical data is the cornerstone for building intelligent prediction models and advancing precision medicine. However, in practical applications, the role of cross-institutional data in risk prediction, outcome assessment, model external validation, and robustness testing is increasingly prominent. In real research and application scenarios, multi-center structured clinical data are often difficult to directly aggregate and share, due not only to privacy protection requirements, but also to multiple constraints such as institutional governance rules, data usage authorization, and compliance reviews. More critically, the privacy sensitivity of data leads to a pervasive “data silo” phenomenon. Since patient information is strictly protected by regulations such as HIPAA, GDPR, and the Personal Information Protection Law, direct data sharing faces significant ethical and legal obstacles^1^, which severely restricts the development of multi-center collaborative research and the full realization of the potential value of medical data.

To address the challenges associated with direct clinical data sharing, researchers have explored alternative approaches such as de-identification, federated learning, and synthetic data generation. However, de-identified high-dimensional datasets may remain vulnerable to re-identification, whereas federated learning enables distributed model training but does not provide portable data assets for unified benchmarking or external validation. Synthetic data have therefore emerged as a promising approach for preserving clinically relevant statistical patterns while reducing the direct exposure of patient-level records^1,2^. Existing tabular synthesis methods include statistical approaches such as Gaussian Copula models, as well as deep generative approaches such as the tabular variational autoencoder (TVAE) and conditional tabular generative adversarial network (CTGAN)^1–3^. In the present study, TVAE, Gaussian Copula, and CTGAN were selected as the representative comparison methods.

Despite these advances, many existing tabular synthesis methods follow a static, open-loop paradigm in which model configurations are determined before generation and are not adaptively updated according to the quality of the resulting synthetic data. This limitation is particularly relevant to medical tabular datasets, which commonly contain heterogeneous variable types, sparse categorical levels, highly skewed distributions, heavy-tailed continuous variables, and complex inter-feature dependencies. More recent diffusion- and transformer-based tabular generators have demonstrated promising performance in general tabular synthesis^4–8^; however, their computational requirements, parameter sensitivity, and stability in small-sample clinical settings require further investigation.^9^ These newer model families were therefore considered as relevant methodological context rather than experimental comparators in the present study. A key remaining challenge is the absence of an adaptive feedback mechanism capable of using statistical fidelity and downstream utility to guide generative-model configuration within a non-convex optimization space.

In response to the triple dilemma of “sample scarcity, feature heterogeneity, and privacy sensitivity,” we propose clean-learn-evaluate-optimize (CLEO), a closed-loop framework for medical tabular data synthesis. Unlike open-loop models, CLEO treats hyperparameter tuning as a sequential decision-making problem^10,11^. At its core, CLEO utilizes a Gaussian Mixture Model (GMM) not as a rigid Gaussian assumption, but as a flexible mixture of *K* components capable of approximating complex, non-Gaussian probability densities^12^. By coupling GMM with a Q-learning-based optimization agent, the framework adaptively explores the trade-off space between statistical fidelity and downstream clinical utility. This automated mechanism is designed to improve the capture of long-tail characteristics in clinical variables while assessing privacy risks through empirical audits.

Before presenting the experimental results, we briefly outline the CLEO workflow. CLEO consists of four interconnected modules: Clean, Learn, Evaluate, and Optimize. The Clean module performs domain-driven preprocessing, including outlier handling and rare-category aggregation. The Learn module trains a GMM-based probabilistic generator on the cleaned clinical table. The Evaluate module assesses synthetic data quality from three perspectives: statistical fidelity, downstream predictive utility, and empirical privacy risk. The Optimize module uses Q-learning to update the generator hyperparameters according to the fidelity and utility feedback. Through this closed-loop design, CLEO transforms conventional one-shot synthetic data generation into an adaptive, feedback-driven synthesis process.

The main contributions of this paper are summarized as follows:

First, this research presents the CLEO closed-loop synthetic framework for medical tabular data, designing a general framework with four core modules: data cleaning (Clean), generative learning (Learn), quality evaluation (Evaluate), and closed-loop optimization (Optimize). This framework is specifically designed for the noise and long-tail distribution characteristics of medical data. Furthermore, a tri-dimensional evaluation architecture is established to provide a comprehensive assessment covering statistical fidelity, machine learning utility, and empirical privacy risk auditing. Notably, while fidelity and utility guide the optimization, privacy assessment is integrated as an independent diagnostic audit using metrics like DCR and NNAA to quantify re-identification risks through post-hoc empirical auditing. We explicitly distinguish these diagnostic measures from formal guarantees like ϵ-Differential Privacy, acknowledging that while CLEO prioritizes clinical utility, it provides empirical evidence of reduced observed re-identification risk by examining whether synthetic records concentrate near real individuals in the feature space. In parallel, the methodology introduces an adaptive reinforcement learning-based optimization strategy that moves beyond the static, “black-box” hyperparameter search characteristic of traditional heuristic methods. By formalizing the synthesis as a Markov Decision Process (MDP), the RL agent learns the deep mapping between generative states and multidimensional quality rewards, enabling the framework to adaptively explore the fidelity–utility trade-off in non-convex parameter spaces, which is difficult for standard heuristic methods to handle effectively. Conclusively, the practical efficacy of the CLEO framework is substantiated through clinical validation on high-dimensional multi-center data. Experimental evaluations using an aneurysm dataset containing complex hemodynamic parameters, morphological features, and clinical history demonstrate that the proposed framework outperforms the evaluated representative reference methods in comprehensive quality and transfer utility within the current dataset. These findings support the potential advantage of the framework in handling heterogeneous medical tables and highlight its possible value as a data augmentation tool for small-sample clinical research.

In summary, the CLEO framework addresses non-convex optimization challenges in high-dimensional medical tables by bridging the gap between raw data purification and adaptive generation, facilitating the translation of statistical fidelity into downstream clinical utility.

## Results

### Comprehensive performance comparison

To validate the performance of the CLEO framework, we conducted a benchmark comparison against three mainstream models: TVAE, Gaussian Copula, and CTGAN^3^. To ensure statistical rigor, utility-related results are reported as the mean ± standard deviation over 15 independent downstream TSTR evaluations, accompanied by hypothesis testing to assess whether the observed performance differences were statistically significant. For statistical fidelity, the metrics in Tables 1 and 2 were computed on the final selected synthetic dataset generated by each method after model selection; therefore, the reported standard deviations for fidelity metrics are 0.0000 by design rather than reflecting repeated stochastic re-estimation. The overall evaluation metric is defined as the Combined Score, which integrates statistical fidelity and downstream utility, as shown in Eq. (1).

1 $${Combined}\,{Score}=0.5\times {Fidelity}+0.5\times {Utility}$$

**Table 1:** Summary of overall comparative experimental results

Method	Combined score	Fidelity	Utility
CLEO (Ours)	0.9232 ± 0.0124	0.9241 ± 0.0000	0.9222 ± 0.0248
Copula	0.8843 ± 0.0133	0.8827 ± 0.0000	0.8859 ± 0.0267
TVAE	0.8437 ± 0.0084	0.8989 ± 0.0000	0.7886 ± 0.0169
CTGAN	0.7476 ± 0.0193	0.6947 ± 0.0000	0.8006 ± 0.0387

Utility-related metrics are reported as mean ± SD over 15 independent TSTR evaluations. Fidelity metrics were calculated on the final selected synthetic dataset for each method, which is why their SD values are reported as 0.0000. *p*-values were calculated via a two-sample t-test comparing CLEO against the best-performing reference method, Gaussian Copula. For the calculation of the Combined Score, please refer to Eq. (1). Therefore, the SD of the Combined Score mainly reflects the variability of repeated downstream TSTR utility evaluations rather than repeated re-estimation of fidelity.

(Mean ± SD).

**Table 2:** Breakdown of statistical fidelity sub-metrics

Method	KS	JS	Corr	TVD	Total fidelity
CLEO (Ours)	0.9052 ± 0.0000	0.9014 ± 0.0000	0.9968 ± 0.0000	0.9141 ± 0.0000	0.9241 ± 0.0000
Copula	0.8514 ± 0.0000	0.8655 ± 0.0000	0.9643 ± 0.0000	0.8737 ± 0.0000	0.8827 ± 0.0000
TVAE	0.8848 ± 0.0000	0.8716 ± 0.0000	0.9629 ± 0.0000	0.8968 ± 0.0000	0.8989 ± 0.0000
CTGAN	0.6472 ± 0.0000	0.8046 ± 0.0000	0.4981 ± 0.0000	0.7979 ± 0.0000	0.6947 ± 0.0000

These fidelity sub-metrics were computed on the final selected synthetic dataset generated by each method after model selection; therefore, SD values are reported as 0.0000. The variability in the Combined Score reported in Table 1 therefore mainly reflects the variability of repeated downstream TSTR utility evaluations rather than repeated re-estimation of fidelity.

*TVD* total variation distance, *KS* Kolmogorov-Smirnov, *JS* Jensen-Shannon.

As summarized in Table 1, CLEO achieves a Combined Score of 0.9232 ± 0.0124, significantly outperforming the second-best comparison method (Gaussian Copula) with a *p*-value of 0.000860 (*p* < 0.01). This indicates that our improvements are not due to random initialization but stem from the robust optimization of the GMM-RL closed-loop. While maintaining the highest Fidelity (0.9241), CLEO shows a substantial lead in Utility (0.9222 ± 0.0248), suggesting that models trained on CLEO-generated data retained comparatively strong discriminative performance for the downstream clinical task. To further analyze the quality of the statistical distribution reconstruction, Table 2 provides a granular breakdown of the Fidelity sub-metrics. CLEO consistently outperforms all existing representative models across all dimensions—TVD, KS, JS, and Correlation structure preservation—indicating improved preservation of variable dependencies in this dataset compared with the evaluated comparison methods.

### Evidence of statistical fidelity and structural consistency

To further validate the quality of the statistical distribution reconstruction, we evaluated the fidelity of the CLEO framework across three levels: correlation structures, high-dimensional manifolds, and univariate marginal distributions.

**Fig. 1:**
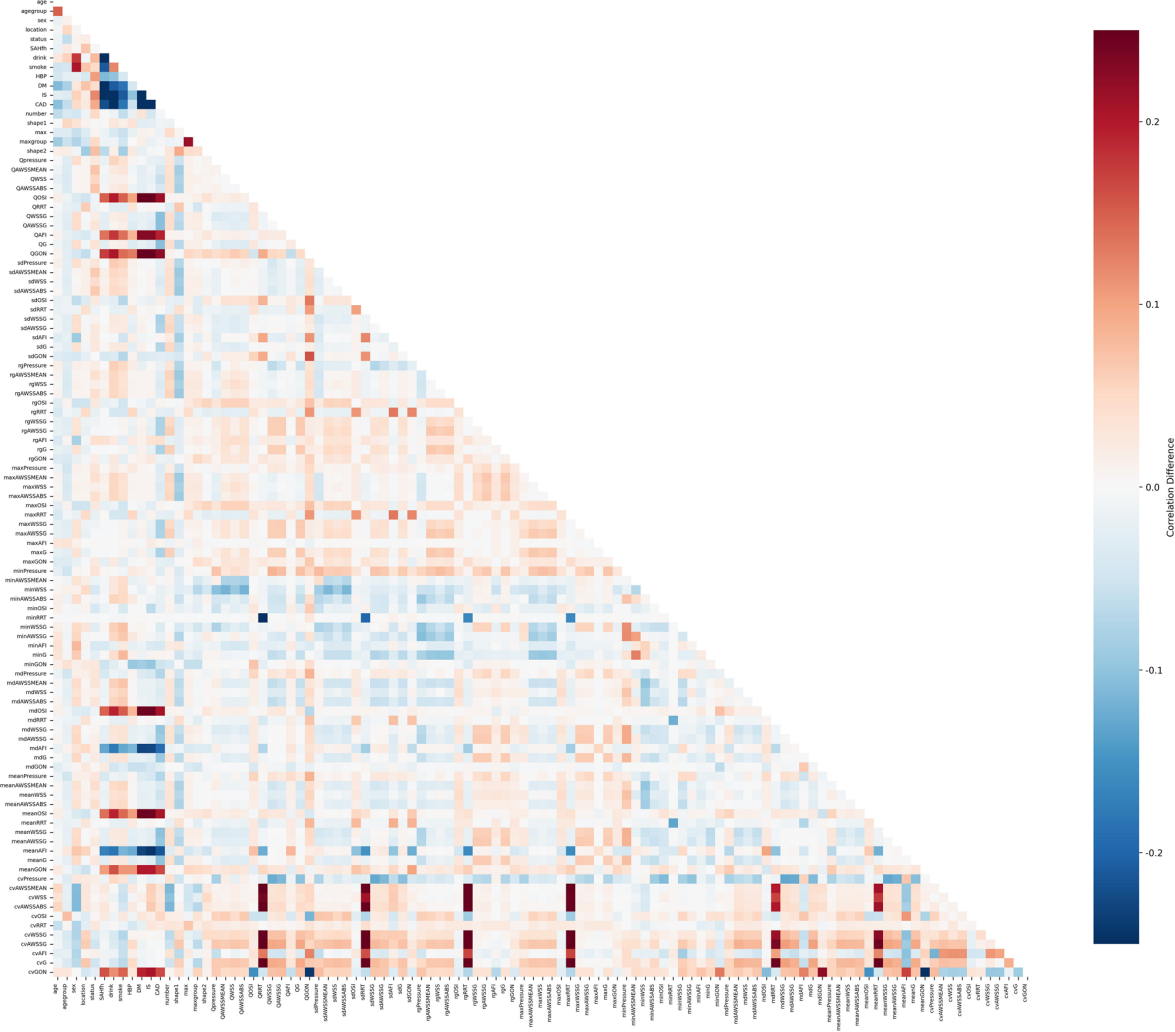
Preservation of correlation structures between real and synthetic datasets. The heatmap illustrates the absolute differences between the Pearson correlation matrices of the real and synthetic data (Diff = C_real − C_syn). Most cells appear close to zero, indicating strong preservation of inter-feature relationships, while even complex hemodynamic interactions remain within a statistically acceptable deviation range of approximately ±0.2.

To evaluate Structural Dependency Preservation, we analyzed the intricate inter-feature dependencies critical for maintaining the clinical logic of synthetic data. As summarized in the Fidelity sub-metrics (Table 2), CLEO achieves a correlation preservation score S_Corr of 0.9968 ± 0.0000. This highly consistent score is visually substantiated by the correlation difference heatmap shown in Fig. 1. Even for highly nonlinear hemodynamic interactions, the correlation differences remain within a statistically acceptable range of ±0.2. This demonstrates CLEO’s superior ability to replicate the complex variable dependencies inherent in multi-center clinical data.

**Fig. 2:**
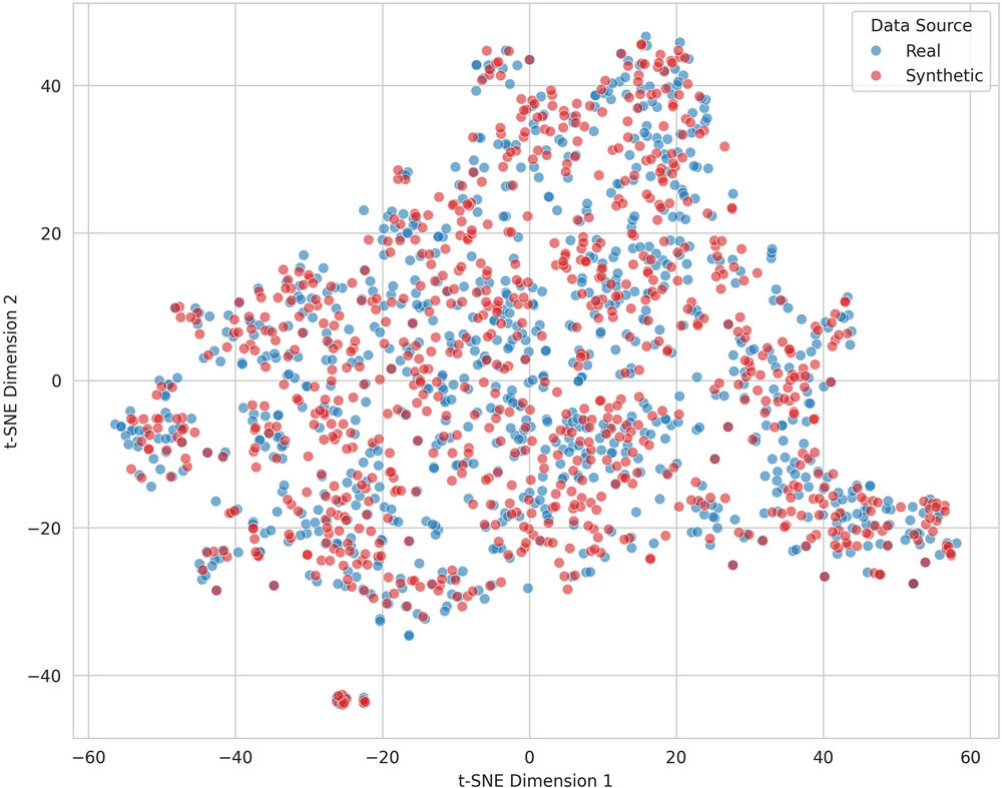
Global distribution coverage of real and synthetic samples visualized by t-SNE projection. The two-dimensional embedding represents the preprocessed clinical feature representation, where blue points correspond to real patients and red points represent synthetic samples. The overlap between real and synthetic samples suggests that the synthetic data broadly covers major regions of the real-data embedding. This visualization provides qualitative evidence of distributional overlap, but should not be interpreted as a formal test for mode collapse.

For High-Dimensional Distribution Coverage, we assessed the global distribution morphology by employing the t-SNE algorithm to project the preprocessed feature representation into a two-dimensional embedding, as shown in Fig. 2. The visualization shows that synthetic samples (red points) and real samples (blue points) are broadly well-mixed, with the synthetic distribution covering the major regions of the real-data manifold. Notably, no clear isolated synthetic clusters or obvious uncovered real-data regions were observed in this projection. This provides visual evidence that the GMM-RL generative process captured the major manifold structure in the evaluated dataset, although t-SNE visualization should not be interpreted as formal proof of the absence of mode collapse or physiological invalidity.

**Fig. 3:**
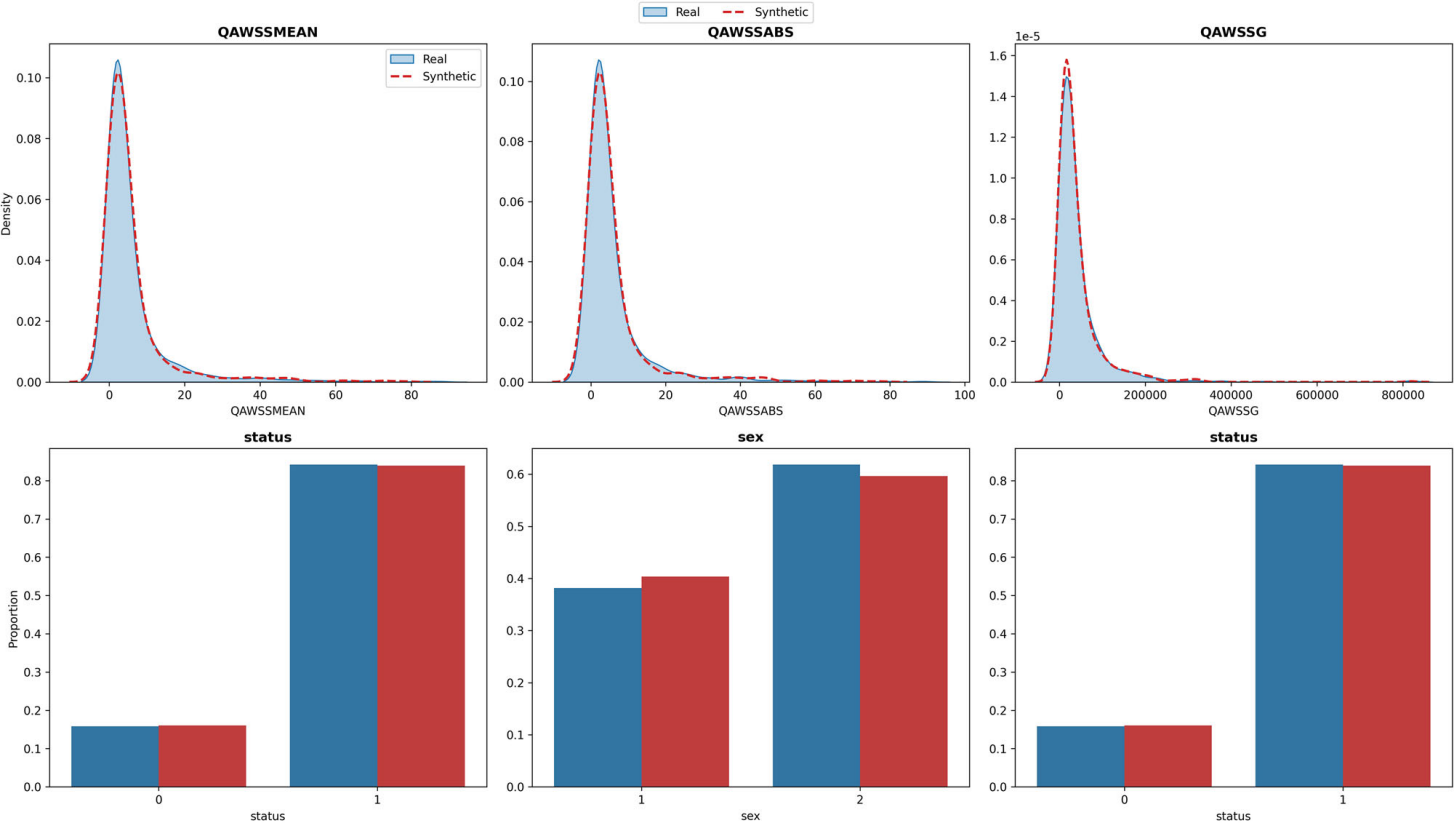
Comparison of univariate statistical distributions between real and synthetic data. The upper panels show Kernel density estimation (KDE) curves for representative continuous variables (QAWSSMEAN, QAWSSABS, QAWSSG), while the lower panels illustrate frequency distributions of categorical variables (status and sex). The close alignment between synthetic distributions (red dashed lines) and real data (blue solid lines) demonstrates accurate reproduction of both central tendencies and long-tail characteristics.

**Fig. 4:**
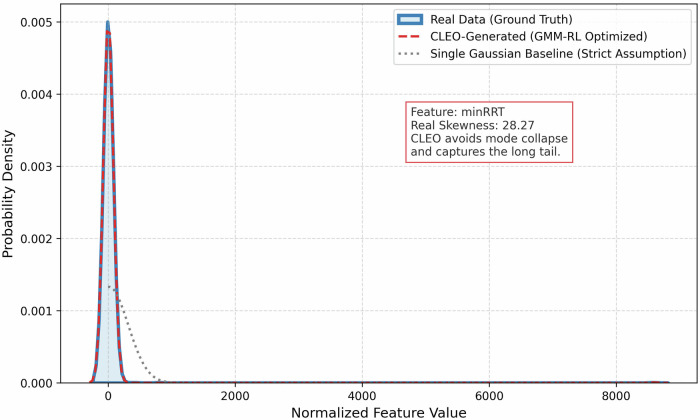
Non-Gaussian distribution fitting performance for the highly skewed feature minRRT. Density curves compare the distribution learned by the CLEO-optimized Gaussian Mixture Model with a single Gaussian reference method. The CLEO model closely matches the real distribution and substantially reduces the Jensen–Shannon divergence (JSD), demonstrating its capability to capture extreme skewness and heavy-tailed clinical data.

Regarding Univariate Density and Skewness Accuracy, we addressed the challenge of heavy-tailed and highly skewed distributions in medical tabular data, particularly in hemodynamic parameters. As shown in Fig. 3, we compared the Kernel density estimation (KDE) curves for continuous indicators and frequency distributions for categorical features. The synthetic contours (red dashed lines) exhibit a high degree of overlap with the real data (blue solid lines). Quantitatively, this is reflected in the strong performance across TVD (0.9141), KS (0.9052), and JS (0.9014) metrics. Specifically, for hemodynamic metrics such as WSS (QAWSSMEAN) and OSI (QAWSSABS), CLEO accurately captures both the sharp peaks and the long-tail features. These results indicate that CLEO effectively captures both sharp peaks and long-tail features in key hemodynamic variables. To specifically evaluate CLEO’s capability in handling non-Gaussian distributions, we performed a comparative fitting analysis on the hemodynamic feature minRRT, which exhibits extreme positive skewness^12^ (28.2711). As illustrated in Fig. 4, while a single Gaussian reference method fails to capture the sharp peak and completely misses the long-tail characteristics, the CLEO-generated distribution shows a substantial overlap with the real data. Quantitatively, as summarized in Table 3, CLEO achieved a Jensen-Shannon Divergence (JSD) of 0.003516, representing a 99.01% reduction in fitting error compared to the single Gaussian model (JSD = 0.356946)

**Table 3:** Quantitative comparison of Non-Gaussian distribution fitting for feature minRRT

Model	JSD (↓)	Skewness handled	Error reduction
Single Gaussian	0.3569	Poor	–
CLEO (GMM-RL)	0.0035	Excellent	99.01%

Jensen–Shannon divergence (JSD) is used to quantify the similarity between the real and synthetic distributions of the highly skewed feature minRRT. Lower JSD values indicate a closer approximation to the real data distribution, and Error Reduction is calculated relative to the single Gaussian.

### Detailed downstream utility analysis: clinical prediction performance

**Fig. 5:**
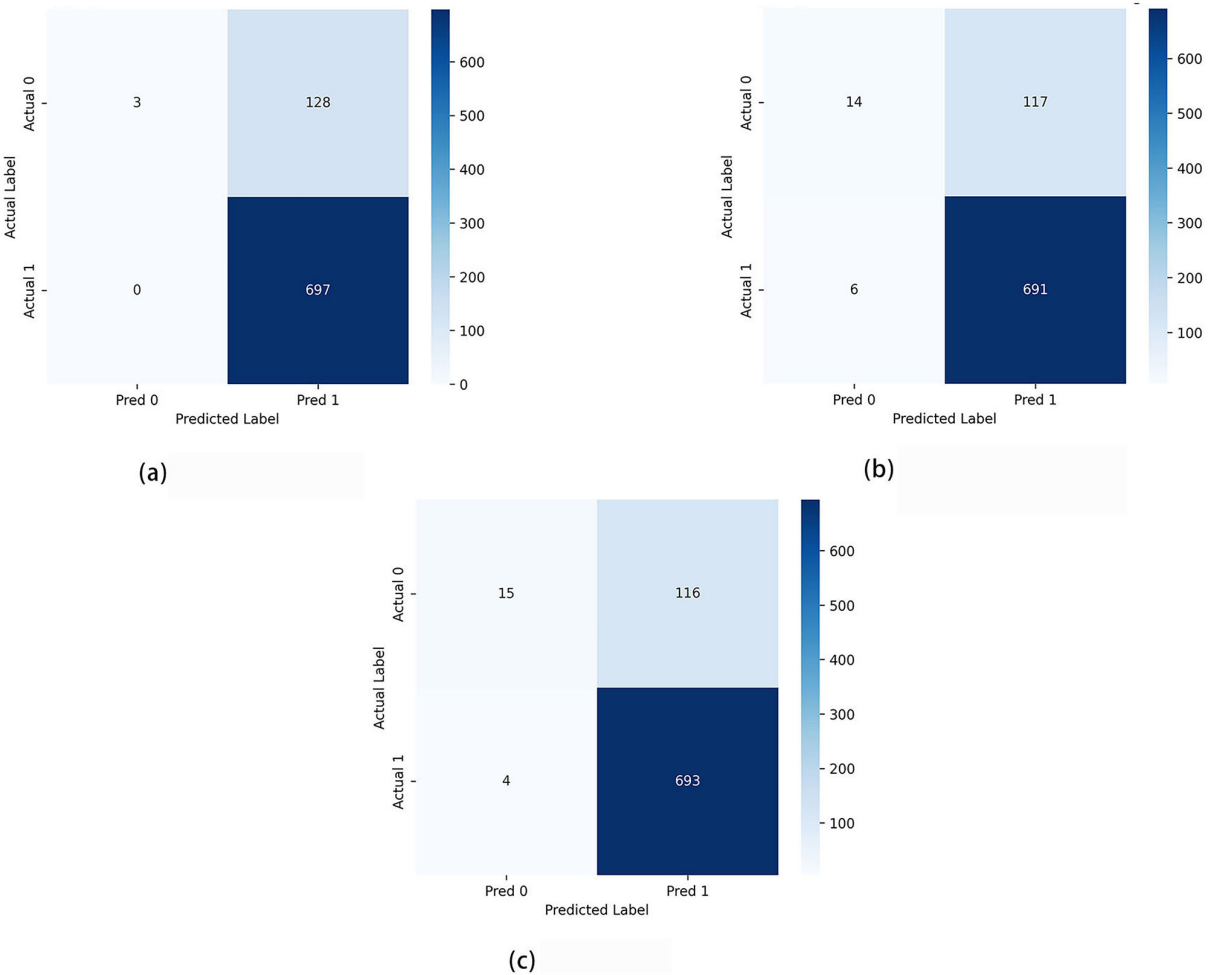
Downstream clinical utility validation using aggregated confusion matrices. The panels present classification results obtained under the TSTR (Train on Synthetic, Test on Real) evaluation paradigm. **a** Random Forest, **b** Gradient Boosting, and **c** XGBoost models trained on synthetic data are evaluated on real test data, indicating that synthetic data retained useful downstream predictive signals in the evaluated TSTR setting, although minority-class prediction remained limited under the imbalanced cohort.

To further evaluate the substitution capability of CLEO-generated synthetic data in practical clinical modeling tasks, we adopted the TSTR paradigm, which is a robust and established evaluation protocol for quantifying the downstream utility of synthetic health data^13,14^. We used “aneurysm rupture risk prediction” as the specific downstream clinical task, comparing the performance difference on an independent real test set between models trained on synthetic data and models trained on real data (TRTR). Considering the characteristics of medical tabular data, we selected three tree-based ensemble learning models that perform best on structured data: Random Forest, Gradient Boosting, and XGBoost. To eliminate the randomness of a single split, we conducted^15^ independent runs with different random seeds and report the average AUC, Macro F1, and standard deviation. As summarized in Table 4, the average AUC across all TSTR models reached 0.7376, suggesting that models trained on synthetic data retained part of the downstream discriminative signal in the evaluated clinical task. Although the average AUC indicates that the synthetic data retained downstream discriminative signals, the relatively low Macro-F1 scores suggest imbalanced performance across classes and should be interpreted in the context of the markedly imbalanced original cohort, as further illustrated by the aggregated confusion matrices in Fig 5.

**Table 4:** Multi-metric prediction performance under the TSTR paradigm

Model	AUC	Accuracy	Precision	Recall	Macro-F1
Random Forest	0.7040 ± 0.0228	0.8454 ± 0.0029	0.8449 ± 0.0026	1.0000 ± 0.0000	0.4799 ± 0.0184
Gradient Boosting	0.7668 ± 0.0384	0.8514 ± 0.0061	0.8552 ± 0.0039	0.9914 ± 0.0084	0.5509 ± 0.0196
XGBoost	0.7421 ± 0.0510	0.8551 ± 0.0110	0.8566 ± 0.0068	0.9942 ± 0.0084	0.5584 ± 0.0406
AVERAGE	**0.7376**	**0.8506**	**0.8522**	**0.9952**	**0.5297**

All models were evaluated under the TSTR (Train on Synthetic, Test on Real) paradigm. Results are reported as mean ± SD based on 15 independent runs with different seeds.

### Privacy protection and re-identification risk assessment

In cross-institutional clinical data sharing, safeguarding patient privacy is the foundational prerequisite. While formal frameworks such as ϵ-differential privacy provide theoretical bounds, their implementation in high-dimensional tabular data often entails a significant utility trade-off. Consequently, this study adopts a multi-metric empirical privacy audit, which is consistent with recent evaluation protocols for synthetic health records. It should be noted that DCR and NNAA are empirical risk indicators rather than formal differential privacy guarantees; therefore, they should be interpreted as evidence of reduced observed re-identification risk rather than as mathematical proof of privacy protection. These metrics assess re-identification risks by analyzing the relationship between synthetic and real samples in the high-dimensional feature space.

**Fig. 6:**
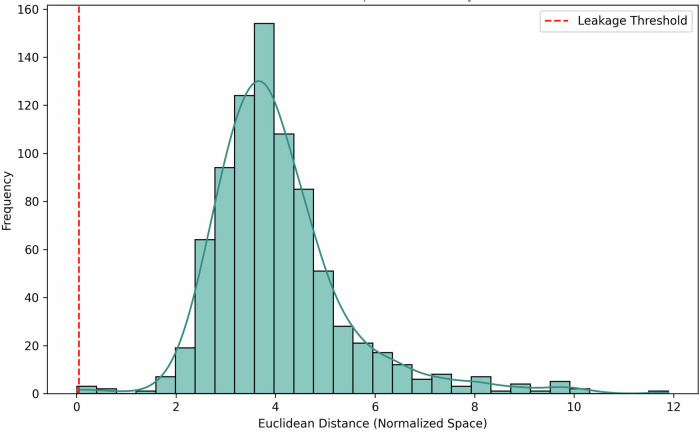
Empirical privacy audit based on distance to closest record (DCR). The histogram shows the distribution of Distance to Closest Record between synthetic samples and real patients in the normalized feature space. The absence of zero-distance records and the right-skewed distribution suggest no obvious exact record replication under this empirical audit setting.

DCR is an empirical metric^16^ for auditing potential sample memorization by measuring the proximity of synthetic records to their nearest real-world counterparts. As demonstrated in the DCR distribution (Fig. 6), the synthetic data exhibits a clear unimodal right-skewed pattern with a mean distance of 4.0624 and zero exact matches. This empirical evidence suggests that the CLEO framework captures global distributional patterns without obvious exact record memorization in the evaluated dataset, indicating reduced observed risk of direct identity leakage.

To empirically audit membership-inference risk, we adopted the NNAA metric, a nearest-neighbor-based adversarial auditing paradigm used in privacy-preserving synthetic data evaluation. Under this framework, an adversary is assumed to use nearest-neighbor information to infer whether specific individuals from the original cohort are present in the synthetic dataset. CLEO achieved a nearest neighbor adversarial accuracy (NNAA) score of 0.4940, which is close to the ideal value of 0.5, representing random guessing under this audit setting. This result suggests low observed membership-inference signal in the evaluated feature space. However, it should be interpreted as empirical audit evidence rather than a formal privacy guarantee.

### Ablation study: module contribution analysis

**Fig. 7:**
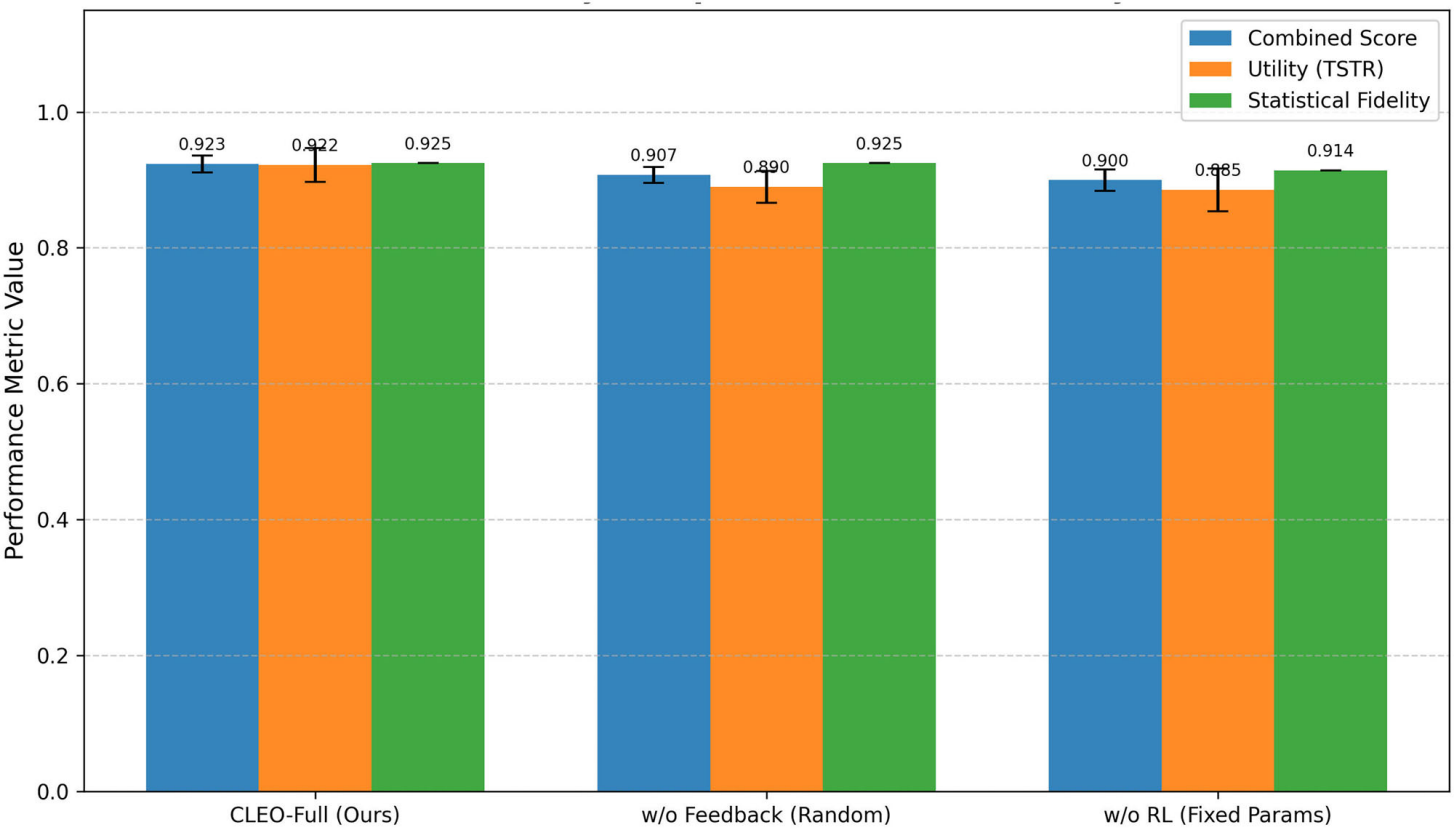
Ablation study demonstrating the contribution of each CLEO module. Bar plots compare the Combined Score, Utility (TSTR performance), and Statistical Fidelity across the full CLEO framework and its ablated variants. Error bars represent the standard deviation across 15 independent runs, illustrating the stability of the framework and the performance improvement introduced by reinforcement learning optimization.

CLEO-Full (Ours) represents the complete implementation of the framework. To evaluate the contribution of each component, we configured three variants. The first, w/o Feedback (Random), replaces the reinforcement learning (RL) closed-loop mechanism with a Random Search strategy to assess the efficiency of the Q-learning agent. The second, w/o RL (Fixed), removes the Optimize module and uses empirically fixed GMM hyperparameters. The third, the reference method (Pure GMM), employs a standard GMM with default parameters on raw data. Utility-related results are reported as mean ± SD over 15 independent TSTR evaluations with predefined random seeds. Fidelity values in the ablation study were computed on the final selected synthetic dataset for each variant after model selection; therefore, Fidelity SD values are reported as 0.0000. By comparing these variants, we reveal the cumulative contribution of data cleaning and closed-loop optimization to synthetic data utility, with the quantitative results summarized in Table 5. Figure 7 (Ablation Study: Component Performance Analysis) visually presents the score distribution across three key dimensions: Combined Score (blue), Utility (orange), and Statistical Fidelity (green). The error bars (Standard Deviation) clearly demonstrate the stability of the CLEO framework across 15 trials. As shown, while all variants maintain a high Fidelity plateau (≈0.92), a significant performance leap is observed in the Utility metric. The transition from fixed parameters (0.8853) to Random Search (0.8900) and finally to the RL-optimized CLEO-Full (0.9222) forms a steady ascending staircase. This visual evidence supports the hypothesis that the closed-loop optimization module is critical for enhancing clinical utility while maintaining statistical fidelity.

**Table 5:** Ablation experiment results: comprehensive performance (Mean ± SD)

Variant	Combined score	Fidelity	Utility (TSTR)	Rel. drop (%)
CLEO-Full (Ours)	0.9234 ± 0.0124	0.9246 ± 0.0000	0.9222 ± 0.0248	0.00%
w/o Feedback (Random)	0.9074 ± 0.0118	0.9247 ± 0.0000	0.8900 ± 0.0235	1.73%
w/o RL (Fixed)	0.8997 ± 0.0159	0.9141 ± 0.0000	0.8853 ± 0.0317	2.57%

Fidelity values in the ablation study were computed on the final selected synthetic dataset for each variant after model selection; therefore, Fidelity SD values are reported as 0.0000. Utility and Combined Score variability mainly comes from repeated TSTR evaluations with different random seeds. The marginal difference in Fidelity scores compared with Table 1 is due to the reclassification of quasi-numerical features, such as age group and number, as continuous variables in the ablation study to enable a more granular correlation analysis.

Comparing CLEO-Full (0.9234) with the w/o RL variant (0.8997), we observe a 2.57% improvement in the combined score, primarily driven by a gain in downstream utility. Furthermore, the higher performance of CLEO-Full compared with the Random Search variant (w/o Feedback, 0.9074) suggests that the Q-learning agent can use feedback from previous evaluations to explore the hyperparameter space more efficiently than purely heuristic search in this dataset. These results provide empirical evidence that the closed-loop optimization module contributes to improved downstream utility while maintaining statistical fidelity.

### Optimization dynamics analysis

**Fig. 8:**
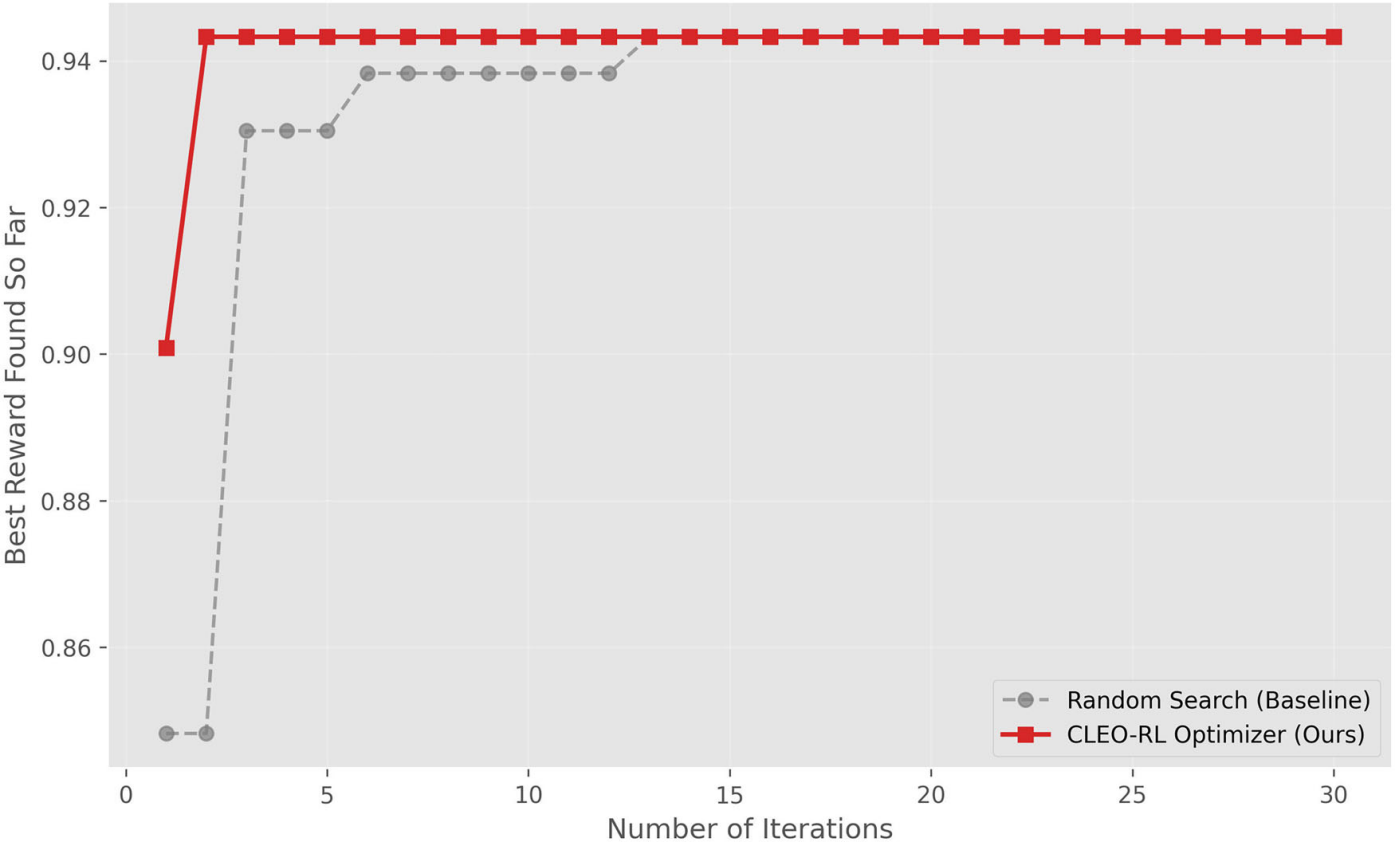
Optimization efficiency comparison between reinforcement learning and random search. The curves display the best reward discovered across 30 iterations for the CLEO reinforcement learning optimizer and a standard Random Search reference method. The faster convergence of the RL agent in this experiment supports the use of sequential decision-making for hyperparameter tuning under the current setting; this comparison is empirical and limited to the evaluated dataset.

To demonstrate the conceptual distinction and efficiency of the proposed closed-loop framework, we conducted a comparative benchmark between the CLEO-RL optimizer and a standard Random Search (RS) strategy over 30 trials. As illustrated in Fig. 8, the CLEO-RL optimizer reached the optimal reward plateau more rapidly than Random Search. While Random Search required 13 iterations to find a comparable configuration, the RL-based strategy converged substantially faster.

### Reward sensitivity and convergence analysis

**Fig. 9:**
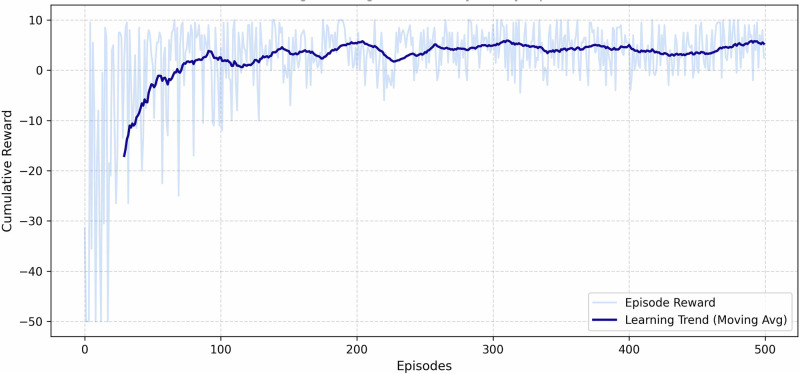
Convergence behavior of the Q-learning optimization agent. The figure plots cumulative reward per episode together with a moving-average trend line across training iterations. The upward trajectory followed by stabilization suggests that the reinforcement learning agent can improve the reward under the tested settings.

**Fig. 10:**
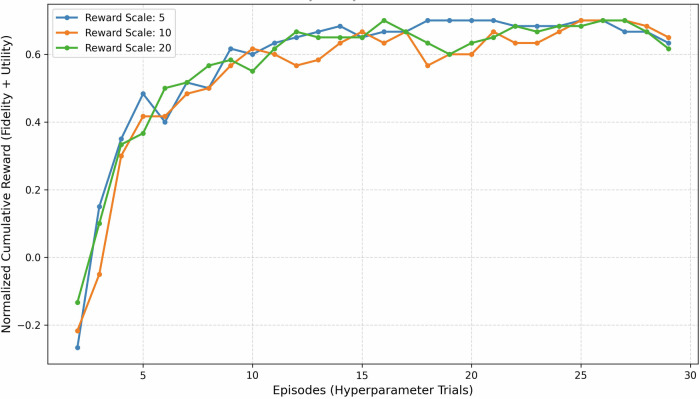
Sensitivity analysis of the reinforcement learning reward scale factor. Reward trajectories are shown for three different scaling parameters (5, 10, and 20). Despite slight differences in early learning dynamics, all configurations converge to similar reward plateaus, suggesting empirical stability of the optimization trajectory under the tested reward scales.

To examine the stability of the reinforcement learning agent under different reward magnitudes, we performed a sensitivity analysis on the reward function by varying the reward scale factor (Scale: 5, 10, 20). In this study, the reward was defined as an equal-weighted combination of the fidelity-related component and downstream utility, rather than as a threshold-triggered sparse reward. The equal weighting was empirically selected to balance distributional similarity and downstream predictive usefulness. The convergence trend shown in Fig. 9 suggests that the agent can learn to improve the fidelity–utility trade-off under the tested settings. As shown in Fig. 10, although the initial learning trajectory varies slightly with different reward scales, all tested configurations converge to a high normalized cumulative reward after approximately 30 episodes. These results should be interpreted as empirical evidence of stability in the current dataset rather than as a formal convergence guarantee.

## Discussion

This study addresses a critical bottleneck in digital health practice: the inherent difficulty of sharing cross-institutional clinical tabular data due to privacy and governance constraints. To circumvent the limitations of traditional open-loop synthesis^1,16^, we propose and validate the CLEO closed-loop framework. In this context, CLEO shifts the design logic from static generative modeling to an adaptive evaluation-optimization loop. Unlike conventional manual hyperparameter tuning or random search methods that often fail to navigate the non-convex objective space of medical synthesis, the CLEO-RL agent formalizes the generative process as a sequential decision-making problem. By modeling the synthesis as an MDP, the RL agent learns the deep mapping relationship between generative states and quality rewards^11,15,17^. This mechanism enables the algorithm to intelligently navigate high-dimensional parameter spaces and leverage complex coupling relationships between clinical features—a behavior that may be difficult to obtain with independent point-search strategies under the same search budget.

From the perspective of methodological mechanisms, the framework first implements domain-driven medical data purification through the Clean module. By adopting the Local Outlier Factor (LOF) algorithm—a density-based approach—rather than the global 3*σ* rule, the framework preserves clinically significant extreme case subgroups while effectively handling statistical noise. This is complemented by a Rare Category Aggregation (RCA) strategy that addresses the long-tail distribution of discrete features, thereby reducing the impact of high-dimensional sparse spaces on generative stability and providing standardized input data for the model.

The evaluation architecture of CLEO is established as a tri-dimensional system covering fidelity, utility, and empirical privacy auditing. In the Fidelity dimension, the framework integrates metrics such as TVD, KS test, and JS divergence to quantify distribution similarity and correlation structure preservation. The Utility dimension adopts the Train on Synthetic, Test on Real (TSTR) paradigm^13,14,18^, applying a Robust Aggregation strategy to provide stable feedback signals for the optimization process by calculating the median of repeated experiments. The Privacy dimension serves as an independent empirical diagnostic audit rather than a formal protection guarantee. By employing empirical privacy indicators such as distance to the closest record (DCR) and NNAA^19–21^, CLEO provides evidence that the generated data does not show obvious exact memorization or strong nearest-neighbor membership-inference signals in the evaluated feature space.

The relatively low Macro-F1 scores observed in Table 4, particularly for the Random Forest classifier (Recall = 1.0000, Macro-F1 = 0.4799), reflect the highly imbalanced class distribution in the dataset. This indicates that while classifiers trained on CLEO-generated data captured the dominant class pattern, minority-class prediction remained challenging. This behavior is consistent with the fact that CLEO currently aims to preserve the original data distribution rather than actively rebalance minority classes. Therefore, the TSTR results should be interpreted as evidence that CLEO preserves major downstream discriminative signals, rather than as evidence of robust minority-class prediction.

In contrast to the inherent limitations of a single Gaussian distribution in capturing the heavy-tailed and skewed patterns of clinical data, the GMM functions as a universal approximator capable of representing any continuous probability density. As substantiated by Scrucca^12^, the flexibility of GMMs stems from the linear combination of multiple components, which allows the model to approximate complex, non-Gaussian distributions that a single Gaussian fails to represent. Within the CLEO framework, this capacity is further amplified by our RL-based agent, which dynamically optimizes the component count *K* to capture nonlinear pathological logic. Our empirical analysis on the highly skewed minRRT feature (Skewness: 28.27) demonstrates that CLEO achieves a 99.01% reduction in fitting error (JSD) compared to a single Gaussian reference method, providing empirical evidence that the GMM-based component can approximate this highly skewed feature in the present dataset. Unlike traditional HPO methods such as Random Search or Bayesian Optimization, which treat each hyperparameter trial as an independent ’black-box’ experiment, CLEO formalizes synthesis as an MDP. The fundamental distinction lies in Policy Learning versus Point Search: while BO seeks an optimal static point, the Q-learning agent learns the relationship between generative states (fidelity/utility) and parameter actions. This enables the agent to exploit the complex coupling between clinical feature dependencies and model capacity. Our results demonstrate that this “informed exploration” allows CLEO to identify the optimal hyperparameter plateau with significantly fewer trials than the reference method. While Random Search requires over 13 independent iterations to locate a comparable configuration, the Q-learning agent effectively navigates the nonconvex objective landscape. This suggests that the RL-based search identified high-value parameter regions more efficiently than random search in the current experiment.

Critically, the privacy dimension of CLEO functions as an independent diagnostic audit rather than a formal mathematical guarantee. We recognize that DCR and NNAA quantify protection within the observed feature manifold and do not preclude theoretical worst-case vulnerabilities. However, in the context of digital health research where data circulation is restricted, this empirical approach provides a practical and auditable safeguard. While CLEO employs DCR and NNAA metrics to empirically evaluate privacy, these are not formal differential privacy guarantees. Similarly, the equal weights used in the reward function were empirically selected to balance statistical fidelity and downstream utility, rather than being derived from a formal theoretical optimization objective. The absence of exact matches (DCR > 0) and the convergence of membership risk to a random reference method suggest that the statistical fidelity of CLEO does not come at the expense of individual exposure.

Overall, CLEO provides a controllable, feedback-driven, and empirically auditable closed-loop framework for synthetic medical tabular data generation under restricted data-sharing conditions. The current results support its feasibility in the evaluated multicenter intracranial aneurysm dataset. Several limitations should be considered when interpreting these findings.

This study has several limitations. First, although the dataset was collected from multiple centers and contains complex morphological and hemodynamic variables, the sample size remains modest and the validation is limited to a single intracranial aneurysm cohort. Therefore, the present findings should be interpreted as empirical feasibility evidence rather than proof of generalizability across all medical tabular datasets. Second, the cohort is markedly imbalanced, and CLEO currently aims to preserve the original data distribution rather than actively rebalance minority classes. This may limit minority-class prediction performance, as reflected by the relatively low Macro-F1 scores. Third, the evaluation protocol focuses mainly on statistical fidelity, TSTR downstream utility, and empirical privacy auditing. Additional analyses, including calibration, fairness assessment, institution-stratified robustness testing, and expert clinical validation, were not conducted in the present study. Fourth, CLEO was compared with representative reference methods including TVAE, CTGAN, and Gaussian Copula, but not exhaustively benchmarked against all recent diffusion- or transformer-based tabular generators. Finally, CLEO does not provide formal differential privacy guarantees; DCR and NNAA should be interpreted only as empirical privacy risk indicators. Future work should validate the framework on larger, multi-disease, multi-institutional datasets and integrate imbalance-aware generation, calibration analysis, fairness evaluation, expert review, and formal privacy-preserving mechanisms.

## Methods

### Study design and ethics

This research was conducted in strict accordance with the principles of the Declaration of Helsinki. This retrospective multicenter study was reviewed and approved by the ethics committees of the participating institutions: the Medical Ethics Committee of Shanxi Provincial Hospital of Integrated Traditional and Western Medicine (Approval No. ZYLLSC-2025-081), the Medical Ethics Review Committee of Hongqi Hospital Affiliated to Mudanjiang Medical University (Approval No. 2025018), and the Biomedical Ethics Review Committee of Shanxi Cardiovascular Hospital (Approval No. 2025xxg 600). The requirement for informed consent was waived by the aforementioned committees. The waiver was granted because the study involved the retrospective analysis of de-identified medical records, posed minimal risk to the participants, and ensured that the rights and welfare of the patients were not adversely affected.

### Data source and research task

This study utilizes an anonymized multicenter clinical database of intracranial aneurysms from three tertiary grade A hospitals in China: Shanxi Provincial Hospital of Integrated Traditional and Western Medicine, Hongqi Hospital Affiliated to Mudanjiang Medical University, and Shanxi Cardiovascular Hospital. The study cohort (D real) comprises 1,035 records of patients with intracranial aneurysms admitted to the participating centers between August 2024 and August 2025. Data extraction and analysis were conducted between August 2025 and December 2025. This cohort includes detailed clinical characteristics and aneurysm rupture status (status: 0 = unruptured, 1 = ruptured), providing a reference benchmark for evaluating synthetic data utility.

The variables are primarily categorized into demographic and clinical baseline information, geometric morphological indicators of the aneurysms, and complex hemodynamic parameters derived from computational fluid dynamics (CFD) simulations. Such a highly complex feature structure is intended to validate the practical efficacy of the CLEO framework in preserving intricate pathological logic and handling data heterogeneity.

To ensure methodological integrity and prevent data leakage, we implemented a standardized preprocessing pipeline. First, basic cleaning was performed by removing insignificant unique identifiers and retaining the candidate predictive variables used for subsequent preprocessing and modeling. Subsequently, we employed a stratified sampling strategy to divide the dataset into independent training, validation, and testing sets at an 80:10:10 ratio (828, 103, and 104 samples, respectively), ensuring consistent class distribution. While the training and validation sets were used for GMM learning and RL-driven optimization, the 10% test set remained strictly isolated as a “blind” benchmark for final utility evaluation under the TSTR paradigm. The detailed distribution is summarized in Table 6. As shown in Table 6, the cohort is modest in size and markedly imbalanced, with the training set containing 131 unruptured and 697 ruptured cases. Therefore, the present experiments should be interpreted as a focused validation on a multicenter intracranial aneurysm cohort rather than as evidence of universal generalizability across diseases, institutions, or clinical tasks.

**Table 6:** Summary of dataset partitioning and class distribution

Data partition	Samples	Class 0 (Unruptured)	Class 1 (Ruptured)
Training set	828	131	697
Validation set	103	16	87
Testing set	104	17	87

The dataset was divided into training, validation, and testing sets using stratified sampling to maintain consistent class proportions across subsets.

### Data preprocessing and quality control

Raw medical data (D real) is first fed into the Clean module for domain-driven data purification to produce high-quality clean data (D clean). Clean Module integrates two purification operations, targeting specific noise patterns for precise processing. It transforms raw medical data (D real) into statistically reasonable and medically correct data (D clean) through a domain-driven purification strategy^22^, thereby improving the stability and reliability of subsequent generative modeling.

This process adopts the LOF algorithm^22,23^. Extreme values in clinical indicators (such as blood pressure, tumor size) may stem from measurement errors or true severe cases, making it difficult to distinguish between the two relying solely on the global 3*σ* criterion. LOF, as an unsupervised density-based algorithm, can avoid global distribution assumptions and identify statistical noise by comparing the local reachability density of a data point with that of other points in its neighborhood (In this study, the number of neighbors was set to 20). In implementation, points with abnormal LOF values (set contamination=“auto”) are identified as local outliers and replaced with the median of that feature.

To alleviate the high-dimensional sparsity caused by discrete variables, we further apply a rare-category aggregation strategy. Discrete features in medical data often exhibit a “long-tail effect,” meaning there are a large number of low-frequency categories. Specifically, all categories with a frequency below a predefined threshold (*N* < 5 in this study) are merged into a unified ‘OTHER RARE’ category. Results show that the feature dimension after encoding with this method is significantly reduced, making the GMM training process more stable. The resulting cleaned dataset is then used as the input to the generative learning stage.

### The CLEO closed-loop synthesis framework

**Fig. 11:**
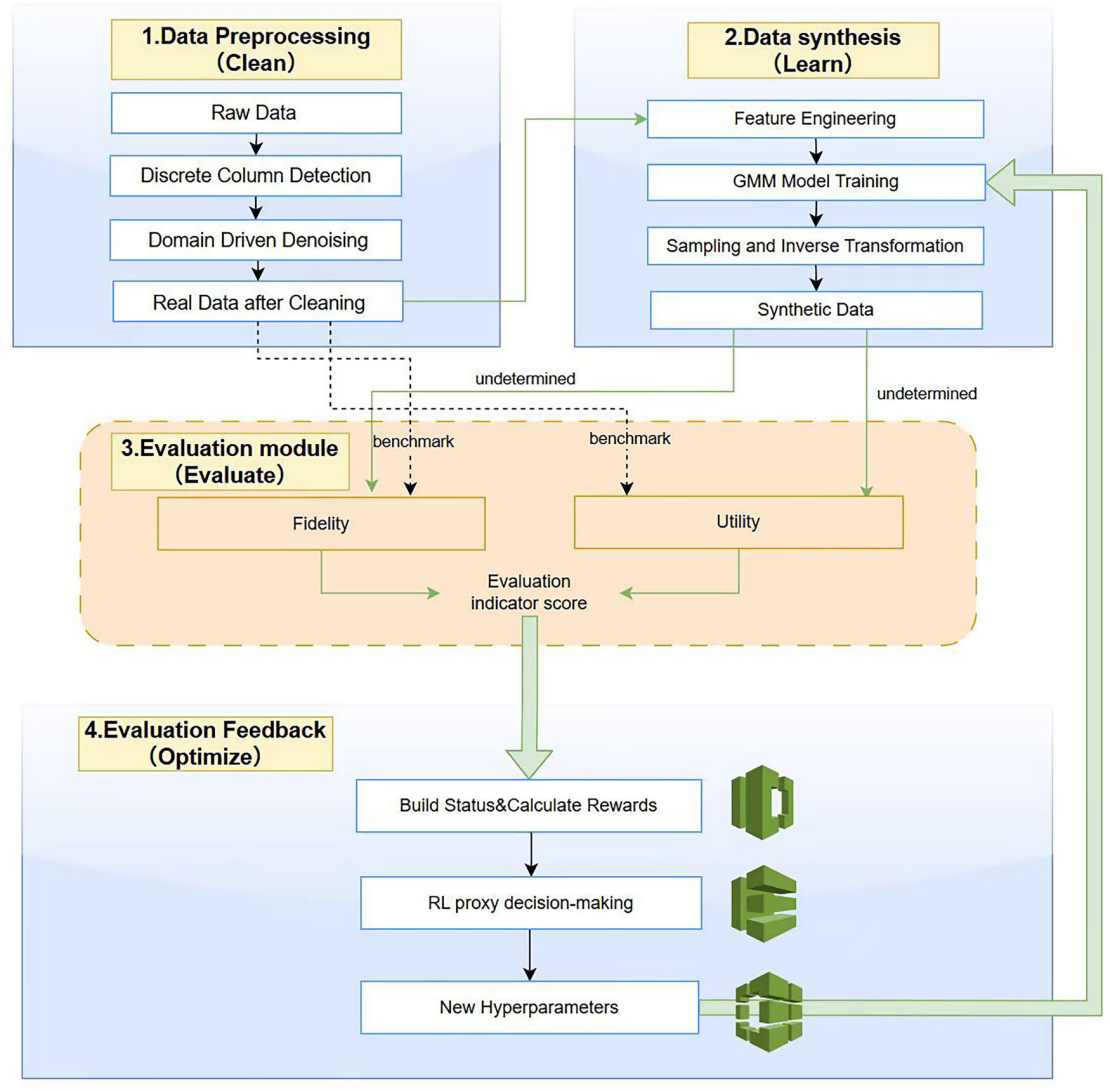
Overall architecture of the CLEO framework. The diagram illustrates the interaction among the four modules: Clean (data purification), Learn (GMM-based generation), Evaluate (tri-dimensional quality assessment), and Optimize (reinforcement learning hyperparameter tuning). The feedback loop enables iterative improvement of synthetic data quality through sequential decision-making.

The core of this project is the CLEO closed-loop optimization framework. Its algorithm architecture, shown in Fig. 11, consists of four core modules (Clean, Learn, Evaluate, Optimize) forming an adaptive intelligent closed-loop system. CLEO formalizes medical tabular data synthesis as a sequential decision-making process in which data purification, generative learning, multidimensional evaluation, and reinforcement learning-based optimization are integrated into a unified feedback loop. While CLEO leverages established components such as GMMs, its methodological contribution lies in the adaptive closed-loop optimization using reinforcement learning, which enables automated tuning of generative parameters based on fidelity and utility feedback, while privacy is assessed through an independent empirical audit. This informed exploration distinguishes CLEO from traditional open-loop synthesis approaches. In the Learn stage, the GMM generator, based on the current hyperparameter configuration *θ*_*t*_, learns the data distribution characteristics of D clean and generates corresponding synthetic data (D syn). As a flexible generative probabilistic model, GMM serves as a universal approximator^12^ capable of accurately modeling the complex, multimodal, and non-Gaussian distributions inherent in clinical tabular data. With a sufficient number of mixture components, the model can flexibly approximate skewed, multimodal, and heavy-tailed clinical distributions. The mathematical principle of GMM assumes that the preprocessed data *x* ∈ R^*D*^ follows a mixture distribution composed of *K* Gaussian components. Its probability density function (PDF) is defined as:

2 $${\rm{p}}({\rm{x|}}\Theta )=\mathop{\sum }\limits_{{\rm{k}}=1}^{{\rm{K}}}{{\rm{\pi }}}_{{\rm{k}}}\cdot {\mathscr{N}}({\rm{x|}}{{\rm{\mu }}}_{{\rm{k}}},{\Sigma }_{{\rm{k}}})$$

where $\Theta =\{{{\rm{\pi }}}_{{\rm{k}}},{{\rm{\mu }}}_{{\rm{k}}},{\Sigma }_{{\rm{k}}}{\}}_{{\rm{k}}=1}^{{\rm{K}}}$ is the model parameter set, *π*_*k*_ is the mixture weight of the *k*-th component $(\mathop{\sum }\limits_{{\rm{k}}=1}^{{\rm{K}}}{{\rm{\pi }}}_{{\rm{k}}}=1){,{\rm{\mu }}}_{{\rm{k}}}$ is its *D*-dimensional mean vector, Σ_*k*_ is its *D*×*D* covariance matrix, and N(·) is the probability density function of the Gaussian distribution.

The model parameters Θ are estimated using the expectation-maximization (EM) algorithm^24^ to find the maximum likelihood estimate for D clean. The EM algorithm iteratively executes the following two steps until convergence:E-step (Expectation): Calculate the posterior probability (“responsibility”) *γ*_*ik*_ that each data point *x*_*i*_ comes from the *k*-th component:

3 $${{\rm{\gamma }}}_{{ik}}=\frac{{{\rm{\pi }}}_{k}{\mathscr{N}}\left({x}_{i},|,{{\rm{\mu }}}_{k},{\Sigma }_{k}\right)}{{\sum }_{j=1}^{K}{{\rm{\pi }}}_{j}{\mathscr{N}}\left({x}_{i},|,{{\rm{\mu }}}_{j},{\Sigma }_{j}\right)}$$

M-step (Maximization): Use the responsibility *γ*_*ik*_ as weights to update the parameters of each component. Let ${N}_{k}\mathop{\sum }\limits_{i=1}^{N}\gamma {ik}$ be the effective number of points for the *k*-th component. The update rules are:

4 $${{\rm{\pi }}}_{k}^{{new}}=\frac{{N}_{k}}{N}$$

5 $${{\rm\Sigma }}_{k}^{{new}}=\frac{1}{{N}_{k}}\sum _{i=1}^{N}{\gamma }_{{ik}}({x}_{i}-{\mu }_{k}^{{new}}){({x}_{i}-{\mu }_{k}^{{new}})}^{T}$$

6 $${\mu }_{k}^{{new}}=\frac{1}{{N}_{k}}\sum _{i=1}^{N}{\gamma }_{{ik}}{x}_{i}$$

The iteration of the EM algorithm guarantees the monotonic increase of the data likelihood function *p*(*X* | Θ) until convergence. After training is completed, sampling a new data point *x*_*new*_ from the GMM is divided into two steps: first, randomly select a component *k* according to the mixture weights *π*_*k*_, then sample a point from the corresponding Gaussian distribution N(*µ*_*k*_,Σ_*k*_). Since the GMM operates on standardized and encoded data, a strict inverse transform must be performed to restore meaningful medical data: for continuous features, apply the inverse transform of the StandardScaler to restore the original scale; for discrete features, apply the inverse transform of the OneHotEncoder to restore the high-dimensional binary vector to a single category. Finally, apply post-processing hard constraints to ensure the generated continuous values are confined within realistic physiological ranges. The synthetic dataset generated in this stage is subsequently examined in the Evaluate module to quantify its fidelity, utility, and privacy characteristics.

The Evaluate module serves as the critical bridge between data generation and closed-loop optimization, responsible for quantifying the quality of the synthetic dataset ${D}_{{syn}}$. To resolve the complex “privacy–utility–fidelity” trilemma inherent in medical data synthesis, this study establishes a tri-dimensional architecture. While the Fidelity and Utility dimensions provide high-signal-to-noise ratio feedback to drive the reinforcement learning agent, the Privacy dimension is integrated as an independent diagnostic audit. This separation ensures that the final generative strategy satisfies rigorous protection requirements without introducing the objective instability often associated with direct privacy-utility optimization trade-offs. In the Fidelity (Statistical Fidelity) dimension, to comprehensively capture univariate distribution characteristics and multivariate dependency relationships, this study constructs a weighted evaluation system containing four complementary metrics. First, the Kolmogorov-Smirnov (KS) test is used to measure the maximum difference in cumulative distribution functions of continuous variables, with the score calculated as *S*_KS_ = 1 − *D*_KS_; second, the Jensen-Shannon (JS) divergence is used to measure the similarity of discretized probability distributions, with the score *S*_*JS*_ = 1 − JS; third, the total variation distance (TVD)^25^ is introduced to quantify the overall difference in probability mass, with the score *S*_TVD_ = 1 − TVD; finally, by calculating the correlation coefficient between the flattened vectors of the Pearson correlation coefficient matrices of real and synthetic data, the preservation degree of inter-feature correlation structure is assessed, denoted as *S*_Corr_. The final Fidelity score is calculated by the weighted sum of the above metrics. Based on the sensitivity differences of each metric to distribution characteristics, the weight configuration is set as: Fidelity = 0.3 · *S*_KS_ + 0.3 · *S*_JS_ + 0.2 · *S*_TVD_ + 0.2 · *S*_Corr_. In the Utility (Machine Learning Effectiveness) dimension, the evaluation focuses on whether the synthetic data preserves the non-linear decision boundaries used for downstream prediction tasks. This study adopts the TSTR evaluation paradigm^26,27^. Specifically, the module integrates three classifiers with different mechanisms: Random Forest, Gradient Boosting, and XGBoost. The system first trains the aforementioned models using synthetic data *D*_*syn*_, then evaluates their prediction performance (AUC metric) on the held-out real test set *D*_*test*_. To eliminate the impact of model random initialization, the Utility score is defined as the mean ratio of the TSTR model’s AUC to the reference method model’s (trained on the real training set) AUC, thus objectively reflecting the substitution level of synthetic data for real predictive capability.

To address the optimization challenges posed by the vast and non-convex hyperparameter space of generative models, the CLEO framework introduces a reinforcement learning (RL)-based Optimize module. This module formalizes the GMM hyperparameter tuning task as a sequential decision-making process, defined as a quintuple $M=\langle S,A,T,R,\gamma \rangle$. Here, $S$ denotes the state space represented by normalized evaluation vectors; $A$ denotes the finite action space consisting of candidate GMM hyperparameter configurations; $T$ denotes the transition induced by retraining the generator and re-evaluating the resulting synthetic data after an action is selected; $R$ denotes the composite reward function based on fidelity and utility feedback; and $\gamma$ denotes the discount factor used in Q-value updating. The corresponding GMM search parameters and Q-learning settings are summarized in Tables 7 and 8. The key RL variables used in CLEO are summarized in Table 9. In this framework, the Optimize module acts as an adaptive search agent that updates GMM hyperparameter configurations based on fidelity and utility feedback, denoted as policy *π*^∗^ by interacting with the environment composed of the Learn and Evaluate modules. Unlike conventional hyperparameter search methods that treat each trial as an independent point search, the RL agent in CLEO learns a policy over the synthesis process. This design enables informed exploration of the parameter space based on the current generation quality rather than blind sampling^10,11,28^. The agent needs to fully perceive the current model’s quality state to make decisions. To avoid computational redundancy from high-dimensional states, we construct the four core evaluation metrics output by the Evaluate module—Total Variation Distance (*S*_*TVD*_), Utility score (*S*_Utility_), KS test score (*S*_KS_), and JS divergence score (*S*_JS_)—into a low-dimensional state vector *s*_*t*_ ∈ S:

7 $${s}_{t}={\left[{S}_{{TVD}},{S}_{{Utility}},{S}_{{KS}},{S}_{{JS}}\right]}^{T},\,{s}_{t}\in {\left[0,1\right]}^{4}$$

where ${S}_{{TVD}}$, ${S}_{{Utility}}$, ${S}_{{KS}}$, and ${S}_{{JS}}$ denote the TVD score, downstream utility score, Kolmogorov-Smirnov score, and Jensen-Shannon score, respectively. This vector is normalized to ensure numerical stability. This design allows the agent to simultaneously capture subtle changes in the model across two orthogonal dimensions: “statistical distribution fitting” and “downstream task utility,” thereby establishing a mapping between hyperparameter configuration and generation quality. The agent’s action *a*_*t*_ ∈ A directly corresponds to a set of key hyperparameter configurations *θ* for the GMM generator. To balance search efficiency and parameter coverage, we define the action space as the Cartesian product of three parameter domains: the number of Gaussian components (K), the maximum number of iterations (M), and the number of random initializations (I):

8 $${\mathscr{A}}={\mathbb{K}}{\mathbb{\times }}{\mathbb{M}}{\mathbb{\times }}{\mathbb{I}}$$

where K = {5,10*,…*,30}, M = {100,200,300}, I = {1,3,5}. This discretized action space contains ${\rm{| }}A{\rm{| }}=6\times 3\times 3=54$ candidate GMM configurations, spanning model capacities from potential underfitting to overfitting. The corresponding GMM parameter ranges are summarized in Table 7, and the Q-learning settings are summarized in Table 8. Because the action space is finite and discrete, Q-learning is used in CLEO as a practical adaptive hyperparameter search strategy rather than as a method for proving global optimality of the non-convex synthesis objective. In the present implementation, the closed-loop optimization ran for 30 episodes, and the complete search process required approximately 20 minutes on a standard workstation. Accordingly, the convergence and reward-sensitivity analyses should be interpreted as empirical evidence of search efficiency and stability in the current dataset, rather than as formal convergence guarantees across all medical datasets. The reward function is the core signal guiding agent evolution. To balance statistical fidelity and downstream utility, we define the base reward rbase as an equal-weighted combination of a fidelity-related component and the utility score. Specifically, the fidelity-related component is calculated as the mean of the TVD score, KS score, and JS score, while the utility component is represented by the downstream TSTR utility score:

9 $${r}_{{base}}=0.5\cdot \left(\frac{{S}_{{TVD}}+{S}_{{KS}}+{S}_{{JS}}}{3}\right)+0.5\cdot {S}_{{Utility}}$$

**Table 7:** Key parameter configuration for GMM probabilistic generative model

Parameter	Symbol	Setting range/value	Description
Number of components	n_components	{5,10,15,20,25,30}	Number of Gaussian components
Maximum iterations	max_iter	{100,200,300}	Maximum EM iterations
Initializations	n_init	{1,3,5}	Random initializations
Covariance type	covariance_type	’full’	Full covariance matrix
Random seed	random_state	42	Base seed for reproducibility

Parameter ranges define the search space explored during reinforcement learning optimization.

**Table 8:** Definitions of key Q-learning variables and hyperparameter configuration

Parameter	Set value	Description
State dimension	4	[*S*_TVD_*, S*_Utility_*, S*_KS_*, S*_JS_]
Action space size	54	6 × 3 × 3 parameter combinations
Learning rate (*α*)	0.1	Controls Q-value update steps
Discount factor (*γ*)	0.95	Balances immediate and future rewards
Exploration decay	0.995	Exponential decay coefficient for *ϵ*
Minimum exploration rate	0.01	Minimum exploration probability

The table summarizes the key state, action-space, and optimization parameters used in the Q-learning-based closed-loop search. These settings define the finite adaptive search space rather than providing a formal global optimality guarantee.

**Table 9:** Definitions of key RL variables in CLEO

Symbol	Definition
S	State space composed of normalized evaluation scores
st	State vector at episode *t*
st = [*S*_TVD_, *S*_Utility_, *S*_KS_, *S*_JS_]	Four-dimensional state vector used by the Q-learning agent
*S*_TVD_	Total variation distance score
S_Utility_	Downstream utility score based on TSTR evaluation
*S*_KS_	Kolmogorov-Smirnov score
*S*_JS_	Jensen-Shannon score
A	Finite action space of candidate GMM hyperparameter configurations
a_t_	Action selected by the agent at episode *t*
θt	GMM hyperparameter configuration corresponding to action at
K	Number of Gaussian mixture components
M	Maximum number of EM iterations
I	Number of random initializations
r_base_(st, a_t_)	Equal-weighted base reward combining fidelity-related scores and utility
R(s_t_, a_t_)	Final reward used for Q-learning update
α	Q-learning rate, set to 0.1
γ	Discount factor, set to 0.95
ε	Exploration rate in the ε-greedy policy

In the implementation used in this study, the final reward directly follows this base reward:

10 $$R({s_{t}},\,{a_{t}})\,=\,{r_{base}}({s_{t}},\,{a_{t}})$$

Here, STVD, SKS, and SJS denote the TVD score, Kolmogorov-Smirnov score, and Jensen-Shannon score, respectively, while S_Utility denotes the downstream utility score. The equal weighting between the fidelity-related component and utility was empirically selected to balance distributional similarity and downstream predictive usefulness. Therefore, the reward function should be interpreted as a practical optimization heuristic rather than a theoretically derived objective. No formal guarantee of global optimality is claimed. To examine whether the optimization trend was overly dependent on reward magnitude, we further conducted a sensitivity analysis on the reward scaling factor.

The Optimize module employs the Q-Learning algorithm^29^ for policy iteration. The agent maintains a Q-value table *Q*(*s,a*) to estimate the long-term expected cumulative reward for taking action *a* in state *s*. In each iteration, the Q-value is updated based on the Bellman Equation using temporal difference learning:

11 $$Q({s}_{t},{a}_{t})\leftarrow Q({s}_{t},{a}_{t})+\alpha [{R}_{t}+\gamma \mathop{max}\limits_{{a}^{{\rm{{\prime} }}}\in {\mathscr{A}}}Q\,({s}_{t+1},{a}^{{\rm{{\prime} }}})-Q\,({s}_{t},{a}_{t})]$$

where the learning rate *α* = 0.1 and the discount factor *γ* = 0.95. To prevent the algorithm from converging prematurely to a local optimum, we implement a dynamic *ϵ*-greedy policy during training. The agent was configured with an ε-greedy action-selection strategy, with the exploration-related parameters summarized in Table 8. :

12 $$\epsilon t=max \left(0.01,1.0\times0.995^{t}\right)$$

Based on the updated state ${s}_{t+1}$ and reward ${r}_{t}$, the RL agent selects the next action ${a}_{t+1}$, corresponding to a new hyperparameter configuration ${\theta }_{\mathrm{t+1}}$.Finally, the new hyperparameters θ*t* _+ __1_ are fed back to the Learn module, replacing the old θt, thus initiating a new round of “generate → evaluate → optimize” iteration. The overall interaction among the four modules is illustrated in Fig. 11.

### Definition of evaluation metrics

The definitions of the fidelity, utility, and privacy metrics are embedded in the Evaluate module above. Table 10 summarizes the corresponding metrics and score formulations for clarity. The present evaluation protocol focuses on statistical fidelity, TSTR-based downstream utility, and empirical privacy auditing because these metrics can be computed reproducibly without exposing patient-level records. Calibration analysis, fairness assessment across demographic subgroups, robustness testing across external institutions, and expert clinical validation were not included in the current protocol and are therefore not claimed as outcomes of this study.

**Table 10:** Evaluation metrics for synthetic data quality assessment

Metric	Description	Calculation/Score
Kolmogorov-Smirnov (KS) test	Maximum difference in cumulative distribution functions of continuous variables	*S*KS = 1 − DKS
Jensen-Shannon (JS) divergence	Similarity of discretized probability distributions	*S*_JS_ = 1 − JS
Total variation distance (TVD)	Overall difference in probability mass	*S*_TVD_ = 1 − TVD
Pearson correlation structure preservation (Corr)	Preservation degree of inter-feature Pearson correlation structure	*S*Corr

The table summarizes the definitions and scoring formulations of the metrics used to evaluate statistical fidelity and structural similarity between real and synthetic datasets.

### Comparative methods and experimental setup

The framework’s efficacy is evaluated through a comprehensive benchmark comparison against established generative models. As detailed in the Comparison of reference method models and parameter settings Table 11, CLEO is compared against deep generative architectures, including TVAE and CTGAN, as well as the statistical Gaussian Copula model. Unlike these static approaches, CLEO utilizes a closed-loop optimization strategy that adaptively searches for optimal parameters over 30 reinforcement learning episodes.

**Table 11:** Comparison of reference method models and parameter settings

Model	Method type	Key parameter settings	Description
TVAE	Deep Generative (VAE)	Epochs: 600	Optimized training epochs for high-dimensional features to ensure convergence
CTGAN	Deep Generative (GAN)	Epochs: 500; Batch Size: 500	Increased batch size to adapt to feature complexity
Copula	Statistical Generative	Default	Standard configuration based on Gaussian Copula function
CLEO	Closed-Loop Optimization (Ours)	Max Iter: 30 (RL Episodes)	Adaptive search for optimal parameters via reinforcement learning

Key parameter settings are listed for TVAE, CTGAN, Gaussian Copula, and the proposed CLEO framework.

### Implementation details and reproducibility

This section details the characteristics of the case dataset used to validate the CLEO closed-loop optimization framework and the key hyperparameter configurations of the generative model and reinforcement learning agent. All experiments were conducted under a unified computing environment to ensure result reproducibility. Regarding the Software Environment and Stochastic Control, the framework was implemented in Python 3.9 using *scikit-learn* for GMM components and *pandas* for data processing. To ensure reproducibility, a base seed of 42 was used for global environment initialization and the RL agent’s exploration. To account for model variance in clinical utility metrics (Mean ± SD), the final TSTR evaluation trials were conducted over a specific sequence of 15 independent random seeds:

$${\rm{S}}=\{13,27,42,77,2025,101,202,303,404,505,606,707,808,909,1010\}.$$

The Execution Workflow is structured as an end-to-end pipeline reproducible via a specific script sequence: first, *split_data.py* performs an 80:10:10 stratified split for training, validation, and testing sets; then, *main_rl_optimization.py* runs the 30-episode RL-driven hyperparameter search for the GMM; and finally, *independent_evaluator.py* generates final fidelity and utility reports on the isolated test set using the 15-seed iteration.

The GMM Generator Configuration involves parameter selection designed to optimize multimodal probability density estimation for high-dimensional heterogeneous data. These settings, including the number of components, maximum iterations, and random initializations, are summarized in the Key parameter configuration for GMM probabilistic generative model Table 7. Similarly, the Q-Learning Optimization Configuration focuses on the parameter settings of the agent to efficiently explore the hyperparameter space and converge to the optimal generation policy within limited training episodes. Detailed values for the state dimension, action space, and learning rates are provided in Table 8. The entire optimization process across 30 episodes takes approximately 20 min on a standard workstation equipped with an NVIDIA GPU.

To ensure the clinical validity and generalizability of the CLEO framework, we implemented a multi-layered rigorous protocol for Methodological Integrity and Leakage Prevention. This includes Strict Stratified Isolation, where the raw dataset was partitioned into training, validation, and testing sets using a stratified sampling strategy to ensure the test set remains an absolute “blind” benchmark held out from the learning and optimization phases. A Preprocessing Firewall was also established to prevent information from validation or test sets from leaking into the synthesis pipeline by fitting all data purification parameters exclusively on the training set. Furthermore, RL Optimization Integrity was maintained by allowing the reinforcement learning agent to utilize only the validation set for calculating rewards and updating the Q-table, ensuring the final performance reflects strictly isolated test data. Finally, Algorithmic Consistency Checks were executed over 15 independent random seeds to demonstrate the stability of CLEO without compromising the integrity of the data split.

## Data Availability

The datasets generated and/or analyzed during the current study are not publicly available because they contain multicenter clinical data subject to ethics and institutional data-sharing restrictions, but are available from the corresponding author on reasonable request and subject to institutional approval.

## Code availability

The code used for data preprocessing, model training, reinforcement learning-based hyperparameter optimization, and evaluation is publicly available at https://github.com/111-abc-111-jpg/CLEOcode.git. Analyses were implemented in Python 3.9 using pandas and scikit-learn. Key model settings and parameter ranges are described in the Methods.
